# A Prospective, Randomized Comparison of Duodenoscope Reprocessing Surveillance Methods

**DOI:** 10.1155/2019/1959141

**Published:** 2019-11-18

**Authors:** Travis J. De Wolfe, Nasia Safdar, Megan Meller, John Marx, Patrick R. Pfau, Eric M. Nelsen, Mark E. Benson, Anurag Soni, Mark Reichelderfer, Megan Duster, Deepak V. Gopal

**Affiliations:** ^1^Department of Biomedical Informatics, University of Pittsburgh School of Medicine, Pittsburgh 15219, PA, USA; ^2^Department of Medicine, Division of Infectious Disease, University of Wisconsin—Madison, Madison 53705, WI, USA; ^3^Infection Control, University of Wisconsin Hospitals and Clinics, Madison 53705, WI, USA; ^4^William S. Middleton Memorial Veterans Hospital, Madison 53705, WI, USA; ^5^Division of Gastroenterology and Hepatology, Department of Medicine, University of Wisconsin School of Medicine and Public Health, Madison 53705, WI, USA

## Abstract

Duodenoscope use in healthcare facilities has been associated with transmission of multidrug resistant pathogens between patients. To assist healthcare facilities in monitoring the quality of their duodenoscope reprocessing procedures and limit patient risk of infection, the Centers for Disease Control and Prevention (CDC) deployed voluntary interim duodenoscope sampling and culturing surveillance protocols in 2015. Though the interim methods were widely adopted, alternative surveillance protocols were developed and implemented at individual institutions. Here, we compared two sampling methods—the 2015 CDC interim protocol and an alternative protocol developed by the University of Wisconsin Hospitals and Clinics (UWHC). We hypothesized that the UWHC protocol would detect a higher incidence of bacterial contamination from reprocessed duodenoscopes. A total of 248 sampling events were performed at UWHC. The CDC protocol (*n* = 129 sampling events) required culturing samples collected from each duodenoscope after brushing its terminal end and flushing its lumen with sterile water. The UWHC protocol (*n* = 119 sampling events) required culturing samples collected from each duodenoscope after swabbing its elevator, immersing its terminal end into broth and flushing its lumen with saline. With the CDC method, 8.53% (*n* = 11) of the duodenoscopes sampled were positive for bacterial growth with 15 isolates recovered. Using the UWHC method, 15.13% (*n* = 18) of cultures were positive for bacterial growth with 20 isolates recovered. The relative risk of identifying a contaminated duodenoscope using the CDC interim method, however, was not different than when using the UWHC protocol. Mean processing time (27.35 and 5.11 minutes, *p* < 0.001) and total cost per sample event ($17.87 and $15.04) were lower using the UWHC method. As the UWHC protocol provides similar detection rates as the CDC protocol, the UWHC method is useful, provided the shorter processing time and lower cost to perform.

## 1. Introduction

Duodenoscopes are flexible, tubular medical devices used by clinicians during endoscopic retrograde cholangiopancreatography (ERCP) procedures. These devices have a distal end which consists of a forceps elevator, elevator recess, and camera, enabling clinicians to examine the pancreatic and bile ducts during an ERCP. Due to their complex design, fragile material composition, reusability, and contact with patient mucous membranes, duodenoscopes are considered semicritical devices that require high-level disinfection (HLD) under the Spaulding classification [1, 2].

HLD is defined as the use of chemical sterilant at short exposure times for inactivation of microorganisms and occurs during reprocessing of the duodenoscope to render it fit for a subsequent use [3]. Despite adherence to recommended manufacturer reprocessing instructions, recent studies have linked healthcare facility outbreaks of multidrug resistant organisms (MDROs) including carbapenem-resistant Enterobacteriaceae (CRE), *Escherichia coli*, and *Pseudomonas aeruginosa* to duodenoscope reuse [4–6]. It is believed that the intricate design of duodenoscopes permits persistent bacterial contamination despite reprocessing, resulting in the subsequent transmission of MDROs to patients [7–9]. In response to the outbreaks, the Centers for Disease Control and Prevention (CDC) deployed the 2015 interim duodenoscope sampling and culturing surveillance protocols to assist healthcare facilities to monitor the quality of reprocessing procedures [10, 11]. The interim protocol was established for sampling duodenoscopes for bacteria located on the distal end and for collecting samples from the instrument channel (via the instrument port to the distal end).

Though the 2015 CDC interim method was widely adopted by healthcare facilities and was successful at recovering contaminating organisms, alternative surveillance protocols were developed at individual institutions [12]. The University of Wisconsin Hospitals and Clinics (UWHC) developed an alternative method to assess duodenoscope reprocessing which included an alternative approach to sample the duodenoscope distal end. It was anticipated that by submersing the distal tip in broth media, recovery of bacteria from the articulations would be maximized. Unlike the 2015 CDC interim method, which was eventually standardized in 2018, the UWHC protocol has not yet been formally evaluated [13].

To offer insight into different duodenoscope surveillance methods, our research group undertook a prospective, randomized comparison of two procedures: the CDC's interim surveillance protocol and the UWHC surveillance protocol. We hypothesized that the UWHC protocol would detect a higher incidence of bacterial contamination from reprocessed duodenoscopes. Finally, we assessed the amount of labor and financial burden associated with implementing either surveillance method.

## 2. Materials and Methods

Surveillance of duodenoscope reprocessing was performed at the UWHC Gastrointestinal Ambulatory Procedure Center by a trained member of the research staff (T.J.D. or M.M.) on two Olympus model duodenoscopes: TJF-Q180 V (*n* = 5) and JF-140 F (*n* = 2). After reprocessing using Rapicide PA in a Medivators Advantage Plus system, duodenoscopes were stored fully dry in a dust-free, nonventilated, closed cabinet in a hanging position with the distal end pointing downward. To eliminate protocol selection bias by the research staff, the surveillance protocols were subjected to random sampling with replacement [14]. This predetermined order dictated which protocol the research staff would use on the available duodenoscopes each day and ensured that all duodenoscopes were subjected to both protocols several times during the study period. We estimated a sample size of 248 sampling events would be necessary to detect a 0.2 difference in the rate of false negative between the two protocol sampling methods (*α* level *p*=0.05, power = 90%). In an effort to provide an adequate number of reprocessed duodenoscopes for regularly scheduled procedures at UWHC and reach the minimum sample size, sampling occurred twice weekly on 3-4 duodenoscopes that were available each sampling day regardless of time since prior reprocessing. Prior to sampling, trained research staff donned proper personal protective equipment and disinfected the countertop, gloves, and outer surfaces of the duodenoscope distal end with sterile alcohol prep pads.

### 2.1. CDC Interim Duodenoscope Surveillance Method

**Figure 1:**
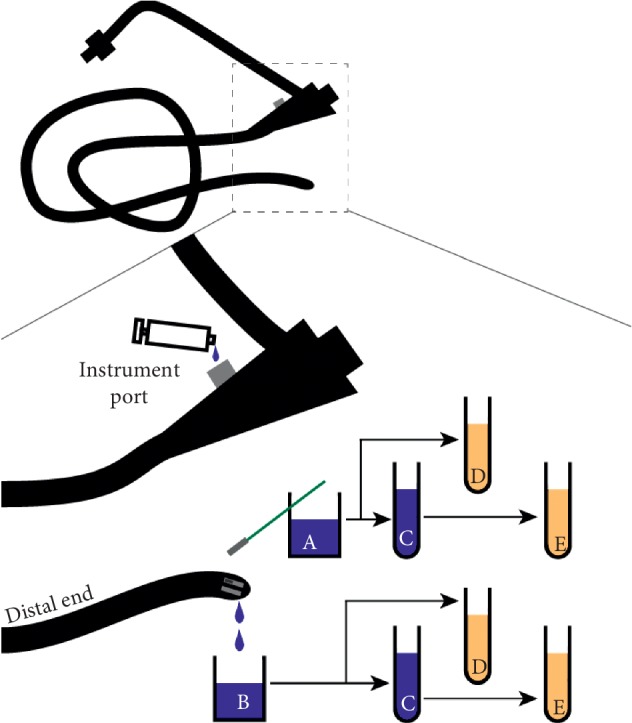
CDC interim duodenoscope surveillance method. (a) The channel cleaning brush is used to sample the distal end of the duodenoscope and placed in a specimen cup containing PBS-T. (b) The syringe containing water is used to flush the instrument channel via the instrument port and collected in a specimen cup. (c) A total of 45 mL from each specimen cup is concentrated via centrifugation. (d) A total of 1 mL unconcentrated specimen is inoculated in 5 mL TSB. (e) A total of 1 mL concentrated specimen is inoculated in 5 mL of TSB. All cultures are subsequently incubated at 37°C for 48 hours.

The CDC's 2015 interim surveillance protocol for sampling was followed as previously described (Figure 1 and [Supplementary-material supplementary-material-1]) [11]. Briefly, duodenoscopes were first adjusted so the elevator mechanism was in a midway position. The handle of a sterile single-use endoscopy ARC channel cleaning brush was shortened by cutting the handle with sterile surgical scissors and then dipped in a prepared sterile specimen cup containing 50 mL of 1X phosphate-buffered saline supplemented with 0.02% Tween 80 (PBS-T). The moistened brush was inserted into and underneath the elevator mechanism with a twisting motion and subsequently used to brush the lens of the duodenoscope. Finally, the brush was enclosed in the sterile specimen cup containing PBS-T used previously and set aside until further processing. A prepared 60 mL sterile syringe containing 50 mL of sterile water was used to flush instrument channel via the instrument port. The flush water was collected into an empty sterile specimen cup. The sampled duodenoscope was subsequently sent for reprocessing.

Immediately after sample collection, the specimen cups containing both the endoscopy channel cleaning brush in PBS-T and the instrument channel flush were agitated with a vortex mixer in ten second bursts for two minutes as previously described (Figure 1 and [Supplementary-material supplementary-material-1]) [10]. While inside a biosafety cabinet, a total of 45 mL from each specimen cup was transferred into individual 50 mL conical tubes and centrifuged (5,000 ×*g*) for 10 min. Any resulting pellets were concentrated by removing 43 mL of supernatant and resuspending the remaining 2 mL volume. Prepared 15 mL conical tubes containing 5 mL of sterile tryptic soy broth (TSB) was inoculated with either 1 mL of the concentrated specimen or 1 mL of the unconcentrated specimen. TSB cultures were incubated at 37°C for 48 hours. Any cultures that developed turbidity were then streaked for isolation on 5% blood agar (BA) and MacConkey agar plates and incubated at 37°C for 24 and 48 hours, respectively.

### 2.2. UWHC Duodenoscope Surveillance Method

**Figure 2:**
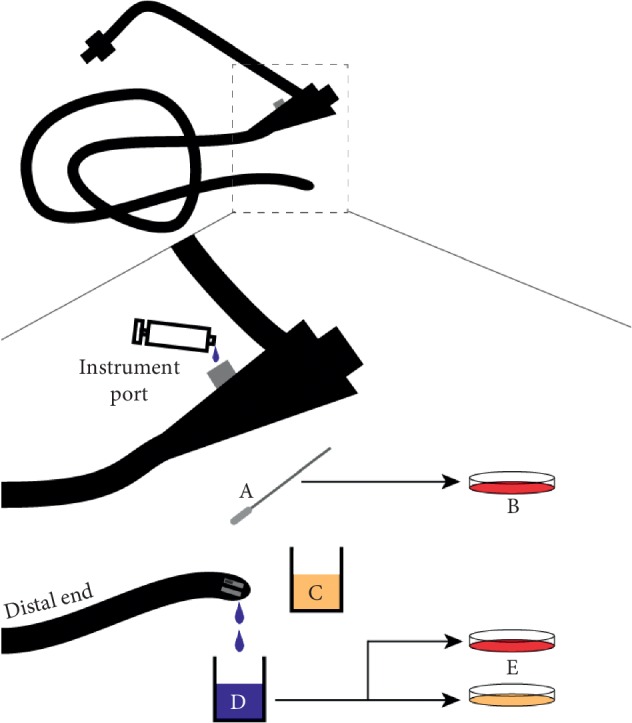
UWHC duodenoscope surveillance method. (a and b) The Dacron swab is used to sample the distal end of the duodenoscope and plated on BA. (c) The distal end of the duodenoscope is immersed and agitated in a specimen cup containing TSB. (d) The syringe containing saline is used to flush the instrument channel via the instrument port and collected in a specimen cup. (e) The collected saline flush is then streaked for isolation onto BA and TSA. All cultures are subsequently incubated at 37°C for 5 days.

A sterile Dacron swab was aseptically moistened in a sterile specimen cup containing 50 mL TSB (Figure 2 and [Supplementary-material supplementary-material-1]). The moistened swab was inserted into and on the elevator mechanism and around the adjoining sides of the elevator cavity. The swab was then immediately streaked onto BA at the sampling location. Next, the outer surface of the duodenoscope distal end was wiped with sterile alcohol prep pads and air dried. The tip was subsequently immersed into the specimen cup containing TSB and swirled for 30 seconds. Finally, the duodenoscope instrument channel was flushed using 10 mL sterile syringe prefilled with 0.9% saline and collected in an empty sterile specimen cup and set aside until further processing. The sampled duodenoscope was subsequently sent for reprocessing.

The cap of the specimen cup containing sterile specimen cup containing TSB that was used previously was loosened to enable gas exchange and then placed at in an incubator at 37°C for five days. Next, 1 mL of the collected saline flush was streaked for isolation onto both BA and tryptic soy agar. Both streak plates were subsequently placed in an incubator at 37°C for five days. If the TSB used for distal end immersion developed turbidity, it was streaked for isolation on BA.

On each sampling day, the research staff recorded the time required to complete the protocols for each individual duodenoscope. Sampling time was defined as beginning at the point of disinfecting the outer surface of its distal end with an alcohol swab and ending at collecting the lumen flush for reach method. Processing time was defined as beginning at the vortex step (CDC) or placing cultures in the incubator (UWHC) and ending at placement of cultures in the incubator. If any of the cultures were positive for bacterial growth, the duodenoscope sampled was deemed contaminated. Surveillance-related expenses were recorded for estimation of protocol-specific financial burden.

### 2.3. Bacterial Characterization

After incubation, cultures were examined for bacterial growth. On each agar plate, individual representatives for each unique morphology were selected, Gram-stained, examined for catalase activity, and viewed under a microscope. Coagulase tests were conducted on all Gram-positive cocci to differentiate *Staphylococcus aureus* (coagulase-positive) from coagulase-negative Staphylococci (CNS). Antibiotic susceptibility tests were conducted on all *S. aureus* isolates using the Kirby–Bauer disk diffusion susceptibility test. Indole and oxidase tests were conducted on all isolates that stained Gram-negative. Each isolate was finally classified as either low- or high-concern according to CDC recommendations [10]. Based on surveillance results from the prior year, which demonstrated absence of CRE, duodenoscopes were not removed from circulation while culture results were pending but were immediately reprocessed for subsequent use [15]. The difference in incidence of bacterial contamination between the CDC and UWHC protocols was estimated using the relative risk ratio in R version 3.5.2 [16]. Culturing and sampling times were compared with the independent Student's *t* test. Comparisons were considered significant if the *p* value was less than 0.05.

## 3. Results

Duodenoscope surveillance by the study team began on January 19, 2016, and concluded on January 10, 2017. During this time, a total of 891 procedures using the duodenoscopes were performed at UWHC, allowing the study team to complete a total of 248 sampling events (CDC, *n* = 129; UWHC, *n* = 119) and collect a total of 615 cultures (CDC, *n* = 258; UWHC, *n* = 357). Each of the 7 duodenoscopes was sampled an average of 35 times (range: 30–43). From the 129 sampling events using the CDC's interim protocol, 8.53% (*n* = 11) were identified to have contamination (Table 1). From those cultures, a total of 15 bacterial isolates were recovered, including CNS, *Bacillus*, *Micrococcus*, and the two high-concern isolates methicillin-sensitive *Staphylococcus aureus* (MSSA) and carbapenem-susceptible *Escherichia coli*. From the 119 sampling events using the UWHC protocol, 15.13% (*n* = 18) were identified to have contamination (Table 1). From those sampling events, 20 bacterial isolates were recovered including CNS, *Bacillus*, *Micrococcus*, and the high-concern isolate *Sphingomonas*. The relative risk of identifying a contaminated duodenoscope using the CDC interim method was 0.56 (95% CI: 0.28 to 1.14) compared to the UWHC protocol.

Of the 15 bacterial isolates recovered using the CDC protocol, 12 were recovered from the brush that was inserted into the elevator cavity and 3 were recovered from the lumen flush (Table 2). Of the 20 bacterial isolates recovered using the UWHC's sampling protocol, 9 were recovered from the elevator cavity swab, 7 were recovered from duodenoscope immersion, and 1 from the lumen flush.

We determined the mean sampling time to be significantly shorter for the CDC protocol (7.28 minutes vs. 8.04 minutes, *p* < 0.05) (Table 3). The mean processing time, however, was significantly longer for the CDC protocol (27.35 minutes vs. 5.11 minutes, *p* < 0.001). Finally, the time required to obtain culture results is shorter for the CDC protocol (24–48 hours vs. 5 days). After evaluating the financial burden between the two protocols, we found that the total sample and culturing costs ($17.87 vs. $15.04/sampling event) were lower for the UWHC protocol than those of the CDC protocol (Table 4).

## 4. Discussion

Despite proper adherence to manufacturer reprocessing recommendations, the complex design of duodenoscopes can allow for persistence of bacteria including MDROs in the distal end [4, 9]. As such, surveillance of duodenoscope disinfection has become a critical quality control step in healthcare facilities who perform duodenoscope procedures. Institution-specific surveillance protocols which arose alongside the CDC's 2015 interim duodenoscope sampling and culturing surveillance protocols include differing steps that may offer greater detection of contaminants in reprocessed duodenoscopes. To gain insight into different duodenoscope surveillance methods, our research group undertook a prospective, randomized comparison of two procedures: the CDC's interim surveillance protocol and the UWHC surveillance protocol.

There are basic methodological differences between CDC and UWHC's surveillance protocols. The CDC protocol requires an endoscopy ARC channel brush to be inserted into and underneath the elevator mechanism using a twisting motion and to subsequently brush the lens of the duodenoscope. The UWHC protocol employs a Dacron swab to be inserted into the elevator mechanism and the surrounding elevator cavity and includes a duodenoscope broth immersion step which is anticipated to efficiently rinse all articulating surfaces of the elevator mechanism and housing. Though incorporated differently, both methodologies include a broth enrichment step during sample processing. The CDC method requires samples to be concentrated by centrifugation followed by inoculation of TSB with concentrate from either the elevator brush or lumen flush. In the UWHC protocol, broth enrichment is the second step and is performed by immersing the distal end of the duodenoscope in sterile TSB with agitation for thirty seconds. Despite the methodological differences between the two protocols, there was no significant difference in the incidence of bacterial contaminant detection. This finding underscores two important points. First, the finding suggests that the UWHC method can be used in place of the CDC interim protocol. The finding also suggests that clinical laboratories can be successful in developing in-house methods for surveillance that may be just as robust as those developed by governmental agencies during outbreak situations.

The majority of the contaminating bacterial isolates from this study were identified through direct culture. In the UWHC protocol, this direct culture included swabbing and broth immersion of the duodenoscope distal end. With the CDC method, manual brushing of the elevator cavity with an endoscopy ARC channel brush recovered nine more organisms than the lumen flush. The majority of the recovered isolates, including CNS, *Bacillus*, and *Micrococcus* genera, are low-concern organisms that likely represent environmental contamination [17]. Recent guidelines from the British Society of Gastroenterology have stated that identification of skin and environmental contaminants on duodenoscopes does not necessarily represent a failure of reprocessing but an issue of duodenoscope recontamination [18]. It is likely that the cultured bacterial isolates are from the environment of the duodenoscope storage space and from duodenoscope handling by the trained research staff or other healthcare workers. Furthermore, since simethicone is not used during duodenoscope procedures at UWHC, retention of bacterial contamination via fluid droplets is not likely [19].

Several steps in which bacterial contamination could be introduced were noted during the current study and stand as limitations to be considered. In both protocols, broth cultures are exposed to the open environment of the sampling room briefly during the sampling period. Though aseptic technique was used during this time, ventilation and lack of a biosafety cabinet in the duodenoscope storage room could have allowed for environmental contamination during sampling. Culturing the elevator cavity with the endoscopy ARC channel brush per the CDC protocol is another step where contamination may have occurred as after use; the protocol requires submersion of the entire brush into a specimen cup containing PBS-T. While caution was taken to avoid contamination at all protocol steps, it is possible the gloves of the trained research staff, which held the brush handle, were a source of contaminating bacteria in those samples [12]. Though these are limitations, sampling in the environment of the duodenoscope storage room represents a realistic setting where duodenoscope surveillance occurs at many institutions, and as such, similar sources of contamination have been previously been identified [12, 20]. These sources of contamination likely do not inhibit our ability to identify clinically relevant contaminants like CRE.

Surveillance-related labor and financial burdens were important considerations for this study. We found that the total sampling and processing time were significantly shorter for the UWHC protocol. A reduction in sampling and processing time can importantly translate into time saved for trained staff. At the UWHC, approximately 6 duodenoscopes are sampled and cultured each month. At this rate, over 25 hours of surveillance-related labor are saved each year using the UWHC protocol. Incorporation of this saved time into the working schedule of healthcare workers may allow them to better adhere to hospital guidelines to prevent MDRO transmission among patients [21].

Similar to surveillance-related labor, the overall cost of sampling and culturing with the UWHC protocol was found to be less than the CDC protocol. The UWHC saves over $200.00 each year when surveilling 6 duodenoscopes per month. As not all institutions have the funds to monitor reprocessing, the UWHC protocol is attractive as it may provide access to undersupported institutions that do not have means to routinely sample and culture duodenoscopes [22]. There is a recent interest in single-use disposable duodenoscopes as an option to address the challenges of cleaning and reprocessing; however, it is unclear if they are a financially viable alternative for many institutions [23, 24]. An important clinical implication associated with the UWHC protocol is that its limited cost may allow for greater fund allocation to improved patient care. This is critical for patients undergoing therapeutic ERCP as an inpatient procedure where quality of life is oftentimes reduced [25].

Though the CDC recommends surveillance of duodenoscope reprocessing, there is no consensus among medical organizations regarding the frequency at which duodenoscopes should be examined [6]. The ASM does not suggest routine culturing of duodenoscopes on the basis that it alone does not lower the risk of pathogen transmission [26]. A recent document published by the FDA recommends that institutions determine their own surveillance frequency [13]. Additionally, it was reported that without surveillance frequency guidelines, only 31% of respondents conducted microbiologic surveillance over the prior year [27]. It is likely the resource-intensive nature of sampling restricts institutional duodenoscope surveillance as it requires trained professionals, specialized supplies, and equipment, coordination with hospital endoscopy units, and additional duodenoscope reprocessing. This stands as a benefit to the UWHC protocol as it requires less labor, time, and financial burden to perform. To this end, it is important to consider that sample processing in our study occurred in a research lab rather than a clinical lab. Many clinical labs may not have the capacity to assume the workload associated with a culture-based duodenoscope surveillance program, thus further challenging the implementation.

There are important limitations of this study to consider. In 2018, voluntary standardized protocols for duodenoscope surveillance sampling and culturing were released as an update to the interim duodenoscope sampling and culturing surveillance protocols evaluated in this study [13]. This update addressed concerns regarding validation of duodenoscope culturing protocols raised in ASM's April 2015 Policy Statement on Culturing of Duodenoscopes [28]. The 2018 protocols differ from the 2015 interim guidelines in that with sampling, the instrument channel requires a flush, brush, and flush method; the elevator cavity is flushed with sterile water and brushed, and samples are collected and then combined. Another important consideration is that the UWHC protocol may not be ideal for facilities that have a history of MDRO transmission or for those who require quarantine of duodenoscopes until final evaluation of culture results. This can be overcome by preferentially sampling duodenoscopes on Fridays, allowing cultures to incubate over the weekend [29]. However, this may not be feasible for clinics with procedures scheduled on weekends and the number of duodenoscopes available may need to be adjusted. In these cases, technologies including the rapid NOW! test have been examined with some success for their ability to detect endoscope contamination with a rapid turnaround time as compared to classical microbiological techniques like those used in the UWHC protocol [30].

## 5. Conclusions

Surveillance of duodenoscopes to ensure disinfection quality is a resource-intensive process, requiring trained staff to carefully sample, evaluate, and interpret results. The process is costly in supplies, lab equipment, and labor. Some important considerations of the UWHC protocol is that it is simple to follow and relatively inexpensive compared to the interim CDC method without compromising organism detection. Though the two methods include different sampling and processing steps, the probability of identifying contamination using either method was not significantly different. We also identified that the UWHC requires less time and resource costs to sample and process each duodenoscope. These advantages make the UWHC protocol an attractive alternative to the CDC's interim guidelines while being able to identify similar incidence of duodenoscope contamination.

Since MDRO transmission among patients due to inadequately reprocessed duodenoscopes remains a serious problem in healthcare facilities, monitoring the efficacy of the disinfection process by either of the methods outlined here can be beneficial. Further research is needed to longitudinally assess surveillance methods which identify contamination and ultimately improve institutional methods of cleaning, disinfection, and storage of duodenoscopes.

## Figures and Tables

**Table 1:** CDC and UWHC duodenoscope culture results summary.

	CDC	UWHC
Total sampling events, *n* (no. of cultures)	129 (258)	119 (357)
Contaminated duodenoscopes, *n* (%)	11 (8.53)	18 (15.13)
Total bacterial isolates recovered	15	20
Identified isolate classifications (*n*)	CNS (8)	CNS (12)
	*Bacillus* (3)	*Bacillus* (6)
	*Micrococcus* (1)	*Micrococcus* (1)
	*E. coli* (1)^*∗*^	*Sphingomonas* (1)^*∗*^
	MSSA (2)^*∗*^	

^*∗*^High-concern organism.

**Table 2:** CDC and UWHC duodenoscope culture technique summary.

	CDC protocol		UWHC protocol		
	Elevator brush	Lumen flush	Elevator swab	Tip immersion	Lumen flush
Positive cultures	10	3	9	7	1
Total bacterial isolates recovered	12	3	9	7	1

**Table 3:** Comparison of surveillance time between the CDC and UWHC protocols.

	Mean (SD)		Mean difference (95% CI)	*t-*statistic (df)	*p* value^a^
	CDC (*n* = 129)	UWHC (*n* = 119)			
Sampling (min)	7.28 (2.61)	8.04 (2.28)	0.76 (−1.38, −0.15)	−2.45 (245.28)	0.015
Processing (min)	27.35 (10.36)	5.11 (2.56)	22.25 (20.39, 24.11)	23.63 (144.77)	<0.001

^a^Independent Student's *t* test.

**Table 4:** Estimated financial burden for surveillance of a single duodenoscope using the CDC or UWHC protocols.

	Vendor	Manufacturer	Catalog no.	Cost/event (USD)
CDC protocol				
Endoscopy AR channel cleaning brush	Surgmed	Batrik Medical	3423030	2.55
PBS-T	Fisher scientific	Seracare Life sciences	54600027	5.06
Sterile specimen cup	Fisher scientific	Fisherbrand	22150240	0.65
60 mL syringe	Fisher scientific	Fisherbrand	14955455	1.20
Sterile water	Fisher scientific	Gibco	15230170	4.46
50 mL conical tube	Fisher scientific	Corning	1443222	1.55
15 mL conical tube	Fisher scientific	Corning	1495949B	2.31
TSB	Fisher scientific	BD	DF0370173	0.10
BA	Fisher scientific	Thermo scientific	R01198	^*∗*^0.00
MacConkey agar	Fisher scientific	BD difco	DF0075171	^*∗*^0.00
				Total: 17.87

UWHC protocol				
Dacron swab	Fisher scientific	BD BBL	L4363000	0.63
Sterile specimen cup	Fisher scientific	Fisherbrand	22150240	0.65
TSB	Fisher scientific	BD	DF0370173	0.24
BA	Fisher scientific	Thermo scientific	R01198	^#^11.24
10 mL syringe	Fisher scientific	Strategic applications	NC0044250	2.09
TSA	Fisher scientific	BD BBL	B12305	0.20
				Total: 15.04

^*∗*^Costs of these items are relevant only when bacterial growth is identified; ^#^costs will increase if bacterial growth is identified in immersion broth.

## Data Availability

The data generated to support the findings of this study are available from the corresponding author upon request.

## Supplementary Materials

Supplementary Materials cdc_supplementary_protocol_1: step-by-step protocol of the 2015 CDC Interim Sampling and Culture Method for the duodenoscope. uwhc_supplementary_protocol_2: step-by-step protocol of the UWHC sampling and culture method for the duodenoscope.
