# Supplementary material for: A Prospective, Randomized Comparison of Duodenoscope Reprocessing Surveillance Methods
Source: Can J Gastroenterol Hepatol. 2019 Nov 18;2019:1959141. doi: 10.1155/2019/1959141 (PMC6885784; doi:10.1155/2019/1959141)
Supplement: Supplementary Materials — cdc_supplementary_protocol_1: step-by-step protocol of the 2015 CDC Interim Sampling and Culture Method for the duodenoscope. uwhc_supplementary_protocol_2: step-by-step protocol of the UWHC sampling and culture method for the duodenoscope. [file 1959141.f1.zip › 1959141.f1/cdc_supplementary_protocol_1.docx]

**CDC Interim Sampling Method for the Duodenoscope – Distal End and Instrument Channel***

This method is for use in the field to sample reprocessed duodenoscopes (after drying) for bacteria specifically located on the distal end; and for collecting samples from the instrument channel (via the instrument port to the distal end). Ideally, two personnel should perform this protocol, where one will hold the duodenoscope (facilitator) and the other person samples (sampler) accordingly. It is important to sample gently, while thoroughly, in order for optimal sampling and maintaining the integrity of the duodenoscope.

1. Using sterile gloves, sanitize the counter where sampling will be performed and the outer surface of the scope tip with a sterile alcohol pad
2. Using a channel-opening brush pre-moistened with PBS-T, use a twisting motion to sample the inside of the elevator mechanism, recess, and channel
3. Scrub the face of the lens with the brush
4. Place brush in specimen container filled with 10 mL PBS-T and tighten the lid
5. Using 50 mL of sterile water, flush the instrument channel via the biopsy valve and collect at the distal end in a specimen container
6. Fill the syringe with air and flush the air through channel to flush out any remaining fluid into specimen container

**CDC Interim Presence/Absence Culture Method for the Duodenoscope – Distal End and Instrument Channel***

This method is to culture bacteria from reprocessed duodenoscopes (after drying) specifically from the distal end and instrument channel.

1. Vortex the samples for 2 minutes in 10 second bursts
2. Aseptically, remove the channel-opening brush
3. Transfer fluid samples (instrument channel flush, channel-opening brush fluid) to 50 mL conical tubes
4. Concentrate by centrifuging the conical tubes on a benchtop centrifuge equipped for high volume suspensions (range: 3,500-5,000 x *g* for 10-15 minutes
5. Remove supernatant without disrupting the pellet to a final volume of 1 mL. If needed, add PBS-T to a final volume of 1 mL and re-suspend
6. Transfer 1 mL sample to TSB (5 mL)
7. Incubate at 37 °C for 48 hours
8. Check and record turbidity at 18-24 hours (overnight) and 48 hours
9. If the sample is turbid, streak broth for isolation onto blood agar and MacConkey agar plates
10. Incubate at 37 °C for 18 hours (MacConkey agar plates) and 48 hours (blood agar plates)
11. Observe plates for bacterial growth
12. Streak colonies for isolation
13. Characterize pure isolates of “low-concern”, which likely represent skin and environmental contaminants, and species of “high-concern”
    1. “Low-concern” bacteria include, but are not limited to, coagulase-negative *Staphylococci*, *Micrococci*, diptheroids, *Bacillus*, and other gram-positive rods
    2. “High-concern” bacteria include, but are not limited to, *Staphylococcus aureus, Enterococcus, Streptococcus,* viridians group, *Pseudomonas aeruginosa, Klebsiella, Salmonella, Shigella,* and other enteric gram-negative *Bacilli*.

*These protocols are interim and have not been validated. They have been superseded in 2018 by the methods described in *Duodenoscope Surveillance Sampling & Culturing: Reducing the risks of infection* [1].

**REFERENCES:**

1. Administration UFD, Prevention CfDCa, Microbiology ASf. Duodenoscope surveillance sampling & culturing: Reducing the risks of infection. 2018.
