# Supplementary material for: A Prospective, Randomized Comparison of Duodenoscope Reprocessing Surveillance Methods
Source: Can J Gastroenterol Hepatol. 2019 Nov 18;2019:1959141. doi: 10.1155/2019/1959141 (PMC6885784; doi:10.1155/2019/1959141)
Supplement: Supplementary Materials — cdc_supplementary_protocol_1: step-by-step protocol of the 2015 CDC Interim Sampling and Culture Method for the duodenoscope. uwhc_supplementary_protocol_2: step-by-step protocol of the UWHC sampling and culture method for the duodenoscope. [file 1959141.f1.zip › 1959141.f1/uwhc_supplementary_protocol_2.docx]

**UWHC Sampling Method for the Duodenoscope**

1. Label all sampling containers with a study specimen number and the seven-digit identification number of the duodenoscope you will sample
2. Don clean non-sterile gloves
3. Clear and disinfect the work surface with a sterile alcohol pad
4. Remove and discard the duodenoscope reprocessing tag which indicates that the duodenoscope is ready for patient use
5. Adjust the elevator mechanism so that it’s midway. Wipe outer surface of distal 6 inches of duodenoscope tip (including the lens) twice with a sterile alcohol pad, being careful to avoid the elevator mechanism
6. Aseptically moisten a Dacron swab by immersing it into a specimen cup containing 50 mL of sterile tryptic soy broth. Swab the surface of the elevator and adjoining sides of the cavity into which the elevator retracts. Do not swab the lens as it is contiguous with external surface and has been exposed to the interior of the duodenoscope storage cabinet. Streak the Dacron swab immediately onto blood agar at the sampling location
7. Thoroughly wipe outer surface of distal 6 inches of the duodenoscope tip, including the lens surface with sterile alcohol pads while avoiding the elevator mechanism. Use at least two sterile alcohol pads to ensure adequate wetting of surface with alcohol. Repeat if gloved hands or other surfaces have contact with the external surface of the duodenoscope
8. Aseptically immerse the distal end of the duodenoscope into the specimen cup containing 50 mL sterile tryptic soy broth and agitate it for 30 seconds. Avoid positioning your gloved hands and forearms directly over the open specimen cup and keep it extended away from your body to avoid accidental contamination
9. Wipe the outer surface of distal 6 inches of the duodenoscope tip with sterile alcohol pads to remove residual broth. Wipe the stainless-steel channel port situated near the duodenoscope handle with an alcohol pad
10. Hold the distal end of duodenoscope in an empty sterile specimen container, avoiding direct contact of gloved hand with container rim. Raise the tip of the duodenoscope and inject 10 mL of sterile saline with a syringe into the stainless-steel channel port. Bring the distal end down into a vertical position and allow saline to drain out of the lumen into an empty specimen cup
11. The duodenoscope is now considered soiled and should be reprocessed prior to use

**UWHC Culture Method for the Duodenoscope**

1. Place the blood agar plate streaked with the Dacron swab in a 37 °C incubator for 5 days
2. Loosen the cap of the specimen cup containing immersion broth and incubate at 37 °C for 5 days.
3. In a biological safety cabinet, plate 1 mL each of saline from the specimen cup containing the lumen wash onto a blood agar plate and tryptic soy agar plate and incubate at 37 °C for 5 days.
4. Cultures with growth after 5 days should be characterized as “low-concern” or “high-concern”
